# *Nicotiana benthamiana* Elicitor-Inducible Leucine-Rich Repeat Receptor-Like Protein Assists *Bamboo Mosaic Virus* Cell-to-Cell Movement

**DOI:** 10.3389/fpls.2017.01736

**Published:** 2017-10-06

**Authors:** I.-Hsuan Chen, Ying-Ping Huang, Ching-Han Tseng, Jian-Tang Ni, Chung-Han Tsai, Yau-Heiu Hsu, Ching-Hsiu Tsai

**Affiliations:** Graduate Institute of Biotechnology, National Chung Hsing University, Taichung, Taiwan

**Keywords:** EILP, ER-targeting, viral movement, BaMV, *Nicotiana benthamiana*

## Abstract

For successful infection, a virus requires various host factors at different stages such as translation, targeting, replication, and spreading. One of the host genes upregulated after *Nicotiana benthamiana* infection with *Bamboo mosaic virus* (BaMV), a single-stranded positive-sense RNA potexvirus, assists in viral movement. To understand how this host protein is involved in BaMV movement, we cloned its full-length cDNA by rapid amplification of cDNA ends. The gene has 3199 nt and encodes a 969-amino acid polypeptide. The sequence of the encoded polypeptide is orthologous to that of *N. tabacum* elicitor-inducible leucine-rich repeat (LRR) receptor-like protein (*NtEILP*), a plant viral resistance gene, and is designated *NbEILP*. To reveal how *NbEILP* is involved in BaMV movement, we fused green fluorescent protein (GFP) to its C-terminus. Unfortunately, the gene’s expression in *N. benthamiana* was beyond our detection limit possibly because of its large size (∼135 kDa). However, *NbEILP* at such low expression could still enhance BaMV accumulation in inoculated leaves. A short version of *NbEILP* was constructed to remove the LRR domain, NbEILP/ΔLRR-GFP; the expression of this deletion mutant could still enhance BaMV accumulation to 1.7-fold that of the control. Hence, the LRR domain in NbEILP is not an essential element in BaMV movement. We constructed a few deletion mutants — NbEILP/ΔLRRΔTMD (without the transmembrane domain), NbEILP/ΔLRRΔCD (without the cytoplasmic domain), and NbEILP/ΔLRRΔSP (without the signal peptide) — to examine whether these domains are involved in BaMV movement. For BaMV movement, NbEILP requires the signal peptide to target the endoplasmic reticulum and the transmembrane domain to retain on the membrane.

## Introduction

*Bamboo mosaic virus* (BaMV) has a flexuous rod-shaped particle (Lin et al., 1977; DiMaio et al., 2015). The genome of BaMV is a single-strand positive-sense RNA of ∼6.4 kb [excluding the 3′ poly(A) tail] and belongs to the *Potexvirus* genus of the family *Alphaflexiviridae* (Lin et al., 1994). The RNA genome comprises the 5′-end 7 methyl-guanosine cap, the 5′ untranslated region (UTR), five open reading frames (ORFs), the 3′ UTR, and a 3′ end of approximately 300 adenylates (Lin et al., 1994; Chen et al., 2005). ORF1 encodes a 155-kDa replicase for viral RNA capping and replication (Li et al., 1998, 2001a,b; Huang et al., 2005; Meng and Lee, 2017). ORFs 2-4 overlap, called triple gene block (TGB), and encode movement-related polypeptides, with 28, 13, and 6 kDa designated as TGBp1, -2, and -3, respectively (Lin et al., 2004, 2006). ORF5 encodes a 25-kDa coat protein (CP) for viral encapsidation, movement, and symptom determination (Lan et al., 2010; Lee et al., 2011; DiMaio et al., 2015). The 3′ UTR of BaMV interacts with viral-encoded replicase and has roles in minus-strand RNA synthesis, polyadenylation, and long-distance movement (Chen et al., 2017).

The spread of plant viruses from the infected cell can be classified into two modes: the short-distance cell-to-cell movement is via plasmodesmata (PD) with the assistance of virus-encoded movement proteins and host factors (Chou et al., 2013), and the long-distance systemic movement being via vascular tissue (Ryabov et al., 1999). The cell-to-cell movement can be tubule–guided, such as with *Cauliflower mosaic virus*, which encodes movement protein to form tubules and directs the virion particles to pass through (Perbal et al., 1993), or non-tubule–guided, such as with *Tobacco mosaic virus*, which encodes movement protein (p30) to increase the plasmodesmal size exclusion limit (SEL) and facilitates viral movement (Wolf et al., 1989). In potexviruses such as *Potato virus X*, TGBp1 is involved in RNA binding, ATPase, helicase activities, increasing the plasmodesmal SEL (Howard et al., 2004), and formatting the PD-transportable ribonucleic protein movement complex. TGBp2 with two transmembrane domains and TGBp3 with one domain are endoplasmic reticulum (ER)-associated proteins (Krishnamurthy et al., 2003; Mitra et al., 2003). TGBp2 and TGBp3 together with CP and TGBp1 form a complex on the ER membrane (Park et al., 2014). The C-terminal tail of BaMV TGBp2 could direct the TGBp1 to localize at the PD (Ho et al., 2017). Furthermore, the active replicase complex with viral RNA is associated with the movement complex (Chou et al., 2013).

The coding capacity of a small viral RNA genome is limited to facilitate the infection and replication cycle; the RNA virus requires different host proteins directly or indirectly to assist its accumulation (Ahlquist et al., 2003; Hyodo and Okuno, 2016; Nagy, 2016). A few host proteins associated with viral RNA or the RdRp complex are involved in BaMV replication such as the chloroplast phosphoglycerate kinase, heat-shock protein 90, and glutathione *S*-transferase U4 (Huang et al., 2017). The host proteins RabGTPase-activating protein 1, serine/threonine kinase-like protein, and casein kinase 2α participate in BaMV movement (Huang et al., 2017).

A group of proteins with a leucine-rich repeat (LRR) motif from tobacco responding to fungal elicitors and playing a role in defense were designated elicitor-inducible LRR receptor-like proteins (EILPs). Similar proteins in tomato called *Cf* proteins confer resistance to the fungus *Cladosporium fulvum* by recognizing its avirulence proteins secreted during infection (de Wit and Joosten, 1999; Joosten and de Wit, 1999; Dixon et al., 2000; Dangl and Jones, 2001). The basal expression of EILP is low and is increased by treatment with elicitors, which implies that EILP is involved in both preexisting and inducible surveillance systems (Takemoto et al., 2000).

We cloned the full-length cDNA of an EILP involved in BaMV movement in *Nicotiana benthamiana* and designated it NbEILP. We also characterized how NbEILP is involved in BaMV movement.

## Materials and Methods

### Plasmid Construction and Gene Expression Knockdown

**FIGURE 1:**
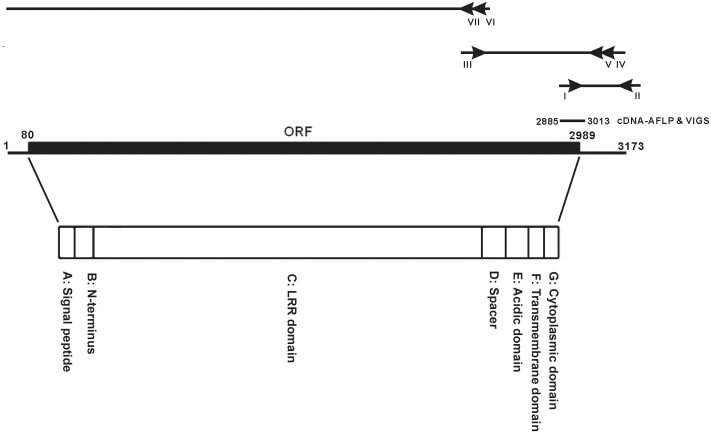
cDNA organization of NbEILP. A diagram to depict the 5′ and 3′ RACE of identifying the sequence of the full-length cDNA of *NbEILP* was illustrated. The primers used in the experiment were shown as I: ACCT8-1-5′/KD (nt 2885–2908), II: XhoI-KpnI-SmaI, III: ACCT8-1/elongation_F (nt 2351–2379), IV: ACCT8-1/5′RACE_R (nt 3109–3081), V: ACCT8-1/5′RACEupR (nt 3048–3020), VI: ACCT8-1/5′RACE2-2 (nt 2503–2476), and VII: ACCT8-1/5′RACE2-n2 (nt 2380–2351). The open reading frame (ORF; nt 80–2989) and cDNA-AFLP fragment (nt 2885–3013) used for VIGS. The encoded polypeptide is divided into the seven domains (A–G) indicated.

The plasmids pTRV1 and pTRV2 were used for *Tobaccorattle virus* (TRV)-based virus-induced gene silencing (VIGS) to knock down host gene expression (Ruiz et al., 1998). The primer set ACCT8-1-5′/KD (5′-CGAACTCCCAACTGGCTTTCTTGG-3′) and ACCT8-1-3′/KD (5′-TAACTCCTCCAGAAGCAAATAGTTTC-3′) was used for PCR amplification of the fragment (**Figure 1**) identified from cDNA-amplified fragment length polymorphism (cDNA-AFLP) (Cheng et al., 2010). The cDNA fragment was cloned into pGEM-T Easy vector (Promega, Madison, WI, United States) and then subcloned into pTRV2 with the *EcoR*I site. The resulting plasmid pTRV2/NbEILP was used to knock down *NbEILP* expression. The negative and positive controls were pTRV2/Luc containing a 398-bp sequence derived from the *Luciferase* gene and pTRV2/PDS containing the phytoene desaturase (PDS) sequence, respectively.

Agrobacteria containing pTRV1 or various pTRV2 constructs were cultured in 1 mL 2YT broth at 30°C overnight, then transferred to 10 mL 2YT broth for growth to OD_600_ = 0.8 ∼ 1.5. After centrifugation at 5000 × *g* for 10 min, cells were re-suspended in induction buffer (10 mM MgCl_2_, 10 mM MES pH 5.7, and 200 μM acetosyringone). pTRV1 was mixed with each TRV2 construct in a 1:1 ratio, then infiltrated onto the second, third, and fourth leaves above the cotyledon of 1-month-old *N. benthamiana* plants.

### Cloning the Full-Length *NbEILP* cDNA

To clone the full-length cDNA of *NbEILP*, 3′ rapid amplification of cDNA ends (RACE) was used to extend the downstream sequence containing the poly(A) tail of the cDNA derived from cDNA-AFLP. A degenerate primer XhoI-KpnI-SmaI-25T-NN (5′-GCTCGAGGTACCCGGGT_25_NN-3′; N: G, A, T, or C) was used for reverse transcription. The specific cDNA fragment PCR-amplified with the primer set ACCT8-1-5′/KD and XhoI-KpnI-SmaI (5′-GCTCGAGGTACCCGGG-3′) was cloned and sequenced. The forward primer ACCT8-1/elongation_F (5′-GAACTTGTTAGAATTTTGACAGTTTACAC-3′) and reverse primer ACCT8-1/5′RACE_R (5′-TATGACAGACATGTCATAAAATAGGGCTG-3′) and a nested reverse primer ACCT8-1/5′RACEupR (5′-CGCCAACATCAACAAATCATTGACATCTG-3′) were used to extend the fragment to 849 bp. For the final step of 5′ RACE, reverse primers ACCT8-1/5′RACE2-2 (5′-GAGTGAGGGTGGTATGCGACCTTGCAAC-3′) and ACCT8-1/5′RACE2-n2 (5′-CGTGTAAACTGTCAAAATTCTAACAAGTTC-3′) were used with the SMARTer RACE cDNA Amplification Kit (Clontech) to extend the sequence toward the 5′ end of the gene.

### Western Blot Analysis

Total protein extracted from plants or protoplasts was separated on 12% polyacrylamide gels containing 0.1% SDS (SDS-PAGE). After separation, the upper portion of the gel containing proteins > 40 kDa including rbcL (RuBisCO large subunit used as a loading control) was stained with Coomassie blue (0.1% w/v Coomassie Brilliant Blue R-250, 50% methanol, 10% acetic acid) and destained with destaining buffer (10% acetic acid, 30% methanol). The stained gel was scanned and quantified by using LI-COR Odyssey. The lower portion of gel containing proteins < 40 kDa including the BaMV CP was transferred onto a nitrocellulose membrane (PROTRAN BA 85 Schleicher and Schnell), which was incubated with rabbit anti-BaMV CP antibody at 4°C for overnight then with anti-rabbit IgG-conjugated IRDye 800 secondary antibody at room temperature for 1 h (Rockland Immunochemicals, Gilbertsville, PA, United States).

For the detection of transiently expressed NbEILP-GFP and its derivatives, the membrane was incubated with the anti-GFP primary antibody, then with the horseradish peroxidase (HRP)-conjugated secondary antibody. The HRP signal (Sensido Advance ECL Substrate kit; Recenttec Inc., Taiwan) was detected by FUJIFILM Image Reader LAS-4000 (Fujifilm Holdings Corp., Japan).

### Northern Blot Analysis

Total RNA harvested from inoculated leaves was denatured in glyoxal buffer (50% DMSO, 1 M glyoxal, 10 mM sodium phosphate buffer) for 1 h at 50°C, separated in 1% agarose gel, and transferred onto Zeta-probe nylon membranes (Bio-Rad Laboratories, Taiwan), which were incubated in 0.2 N NaOH buffer for 30 min (Tsai and Dreher, 1991) and prehybridized with 1× SET buffer (150 mM NaCl, 2 mM EDTA, 30 mM Tris-HCl pH 8.0), 0.1% sodium phosphate, 0.6% SDS, 10× Denhardt’s (2% Ficoll, 2% polyvinylpyrrolidone, 2% bovine serum albumin), 10 mg/ml salmon sperm DNA, 0.25 mg/ml yeast RNAs for 4 h at 65°C. The probe, the 3′-end 0.6-kb BaMV RNA (Lin et al., 1994), was *in vitro*-transcribed from the linear plasmid pBaMV-O/SB2.6 with [α-^32^P]UTP labeling (Huang and Tsai, 1998) and added into the hybridization buffer (same as the prehybridization buffer). After hybridization, the radioactivity on the membranes was determined by using PhosphoImager (Fujifilm BAS 2500, Tokyo).

### Real Time-Quantitative RT-PCR

Approximately 1 μg total RNA was mixed with 1 μl 20 pmole reverse transcription (RT) primer XhoI-KpnI-SmaI-25T-NN and incubated at 70°C for 5 min, then quickly cooled on ice. After mixing, 4 μl of 5x RT buffer, 2.4 μl of 25 mM MgCl_2_, 1 μl of 10 mM dNTP, 0.3 μl RNase inhibitor (Invitrogen, Carlsbad, CA, United States), 1 μl reverse transcriptase (Promega), and deionized water was added to total 20 μl. The mixture was incubated at 25°C for 5 min, then quickly transferred to 42°C for 60 min. Finally, the reaction was stopped by incubating at 70°C for 15 min. qPCR was performed in a 20 μl-reaction containing a 1000X dilution of SYBR green I (Cambrex Bio Science Rockland, Rockland, ME, United States) with 0.6 mM of the primer pair 5′CT18_KD (5′-GAGAAGGCGCAAGAAGC-3′) and 3′/CT18_KD (5′-CAAATGTAAACACATATGACAGAC-3′) in 0.2-ml PCR tubes. The conditions began with an initial denaturation at 95°C for 3 min, followed by 40 cycles of 95°C for 3 s and 60°C for 20 s by use of the KAPA SYBR FAST qPCR Kit (KAPA Biosystems, Boston, MA, United States). Reactions were performed in a TOptical Gradient 96 (Biometra, An Analytik Jena Company, Germany) with data acquisition at 60°C on the channel, excitation at 470 nm and detection at 585 nm. The reaction without template or reverse transcriptase was a negative control, and *actin* was amplified with the primer set actin_5′ (5′-GATGAAGATACTCACAGAAAGA-3′) and actin_3′ (5′-GTGGTTTCATGAATGCCAGCA-3′) for normalization.

### Measurement of BaMV Cell-to-Cell Movement

About 10 μg of the plasmid pCBG (Lin et al., 2004), a BaMV infectious cDNA clone containing the GFP reporter gene and driven by a 35S promoter, was mechanically inoculated onto the fourth leaf above the *Agrobacterium*-infiltrated knockdown leaf of *N. benthamiana*. Green fluorescent lesions on inoculated leaves were observed by fluorescent microscopy (Olympus IX71) at 4 days post-inoculation (dpi). The lesion size was measured by using Image J^1^.

### Construction of *NbEILP*-Deletion Mutants

To construct the LRR domain-deletion mutant, the primer pair BsmIACCT8-1/2643F (5′-GAATGCTTTCAAGGACCTCAATTTGCAAC-3′) and KpnI/ ACCT8-1/2986R (5′-GGTACCTCTTCTGTTGTGCCTTTGACAGGCTC-3′) (sites for restriction enzymes *Bsm*I and *Kpn*I are underlined) was used to amplify the 3′ portion of NbEILP including the spacer, acidic, trans-membrane, and cytoplasmic domains (**Figure 1**). The amplified DNA fragment was cloned into the pGEM-T Easy vector and sequenced.

To construct the transmembrane domain-deletion mutant, two fragments were PCR-amplified with the primer pair BamHI80F (5′-GGATCCATGATGATGGTTCCCAAATTATCTTCTCTTC-3′) and Del_TMD_3′ (5′-TCCAAGAAAGCCATTTCCAGAAATCATTGAG-3′) for the 5′ portion and the primer pair Del_TMD_5′ (5′-TCTCAATGATTTCTGGAAATGGCTTTCTTGG-3′) and KpnI2986R (5′-GGTACCTCTTCTGTTGTGCCTTTGACAGGCTC-3′) for the 3′ portion, with pNbEILP/ΔLRR-GFP used as a template. Two PCR fragments were gel-purified and mixed for another round of PCR amplification with the primer pair BamHI80F and KpnI2986R to generate a 483-bp fragment. The PCR product was cloned and sequenced. The resulting mutant was designated NbEILP/ΔLRRΔTMD.

To construct the cytoplasmic domain-deletion and signal peptide-deletion mutants, the plasmid pNbEILP/ΔLRR-GFP was used as the template with the primer pairs BamHI80F and KpnI_dCD_3′ (5′-GGTACCGTTGGGAGTTCGAGCTGAAAGCATGAAATATG-3′) and BamHI_dSP_5′ (5′-GGATCCATGATGATG TCCACAGAGGAAGCAACTG-3′) and KpnI2986R, respectively. The PCR fragments were cloned and sequenced. The resulting constructs were designated NbEILP/ΔLRRΔCD and NbEILP/ΔLRRΔSP. All mutant constructs in T-Easy vector were digested with *Bam*HI and *Kpn*I and sub-cloned into the expression vector pBIN-mGFP1, with GFP fused at the C-terminus.

### Virus Inoculation and Transient Expression

*Agrobacteria* containing pBIN-NbEILP-mGFP (pBIN61 vector containing NbEILP fused with mGFP), its derivatives (NbEILP mutants) or pBIN-p19 were cultured in 1 mL 2YT broth at 30°C overnight, then applied to 10 mL 2YT broth and grown to OD_600_ = 0.8 ∼ 1.5. After centrifugation at 3500 rpm for 20 min, cells were resuspended in the induction buffer (10 mM MgCl_2_, 0.1 M MES pH 5.7, and 750 μM acetosyringone). Then the *NbEILP*-deletion constructs were mixed with the one containing a p19 silencing suppressor at a ratio of 1:1 at final OD_600_ = 1.

*Nicotiana benthamiana* plants were grown in a growth room with temperature kept at 28°C and 16-h light/8-h dark cycle. Leaves from 3-week-old plants were mechanically inoculated with 50 ng BaMV virion per leaf. At 2 dpi, inoculated leaves were agro-infiltrated with various constructs to transiently express proteins. At 4 dpi (2 days post-infiltration), approximately 0.1 g leaf was collected and smashed into powder with liquid nitrogen.

### Transient Expression of *NbEILP* and Protoplast Isolation

mCherry derived from *Discosoma* sp. (Shaner et al., 2004) is a monomeric red fluorescent protein with peak absorption at 587 nm and emission 610 nm. ER-rk, originally used as an ER marker in the CD3-959 cell line (Nelson et al., 2007) and targeting the ER with red fluorescence, was modified to use as a plant ER marker driven by double 35S promoters and signal peptide in the pBIN20 binary plasmid. The second or third leaf of 3-week-old *N. benthamiana* plants was agro-infiltrated with *NbEILP* or its derivatives with p19 and mCherry in a 1:1:1 ratio.

The preparation of protoplasts from *N. benthamiana* was as described (Tsai et al., 1999). Briefly, approximately 2 g of leaves with transient expression was used for protoplast isolation. The intact protoplasts were collected from the interphase of sucrose and Mannitol-MES after removing the cell wall. Finally, protoplasts were re-suspended in Mannitol-MES after a few steps of washing and stained with fluorescein diacetate to examine the quality and quantity of cells under a fluorescent microscope.

### Cell Extract Fractionation

Approximately 1 g infiltrated leaf at 2 days post-infiltration was homogenized with 2 ml extraction buffer (50 mM Tris-HCl pH 7.6, 15 mM MgCl_2_, and 120 mM KCl, 20% glycerol, 0.1% β-mercaptoethanol, and the protease inhibitor) and centrifuged at 4°C with 400 × *g* for 10 min (Laliberte et al., 2007). The supernatant was collected and centrifuged at 4°C with 21,300 × *g* for 35 min to separate the soluble fraction (supernatant) and the membrane fraction (pellet). The membrane fraction was resuspended in 2 ml extraction buffer. Part of the fractions was mixed with 4X sample buffer (250 mM Tris-HCl pH 6.8, 40% glycerol, 0.02% bromophenol blue, and 10% β-mercaptoethanol) and boiled for 5 min. The proteins were separated on 12% SDS-polyacrylamide gel and subjected to western blot analysis.

## Results

### A Gene Represented by ACCT8-1 Upregulated after BaMV Inoculation Is an EILP

A cDNA fragment, ACCT8-1 (129 bp), identified by cDNA-AFLP, was derived from an upregulated gene after BaMV inoculation in *N. benthamiana* and found to participate in BaMV infection (Cheng et al., 2010). RACE was used to clone the 3′ end of the gene. The 3′-end sequence of the gene was searched in known databases and found similar to a cDNA fragment of a plant resistant gene in *N. tabacum*, CHO_SL009xg14f1.ab1 CHO_SL. Accordingly; a primer (ACCT8-1/elongation F) was designed to extend the sequence up to 849 bp of the gene from *N. benthamiana*. 5′ RACE with 2 reverse primers (**Figure 1**) was used to extend the length of the cDNA to the 5′ end of the gene. The full-length gene is 3173 bp, with 79 and 184 bp of the 5′ and 3′ UTRs, respectively (**Figure 1**). The ORF of this full-length cDNA encodes a 969 amino acid polypeptide (**Figure 1**).

The amino acid sequence is homologous to the EILP gene of *N. tabacum* (Supplementary Figure S1) and the *Cf-2/Cf-5* gene family of tomato (Supplementary Figure S2). ACCT8-1 protein has the same seven domains (A–G) (**Figure 1**) as members of the *Cf-2/Cf-5* family (Supplementary Figure S2). Domain A is a 25-amino acid putative signal peptide for ER entry; domain B is presumed to be the NH_2_-terminus of the mature ACCT8-1 protein; domain C includes all 32 LRRs, whose structure is very similar to that of *Cf-2/Cf-5* and EILP; domain D is a spacer with no known function; domain F is a membrane-spanning region; and domains E and G are the acidic domain and the short cytoplasmic portion rich in basic amino acids, respectively (**Figure 1**). The polypeptide translated *in silico* is very similar to proteins of the *Cf* gene family in tomato that encode the transmembrane receptor-like proteins with extracellular LRRs and a short (23 to 36 amino acid) cytoplasmic domain. Therefore, we designated this gene *NbEILP* (GenBank accession no. MF346698).

### BaMV Accumulation Is Reduced in *N. benthamiana* with *NbEILP* Knockdown

**FIGURE 2:**
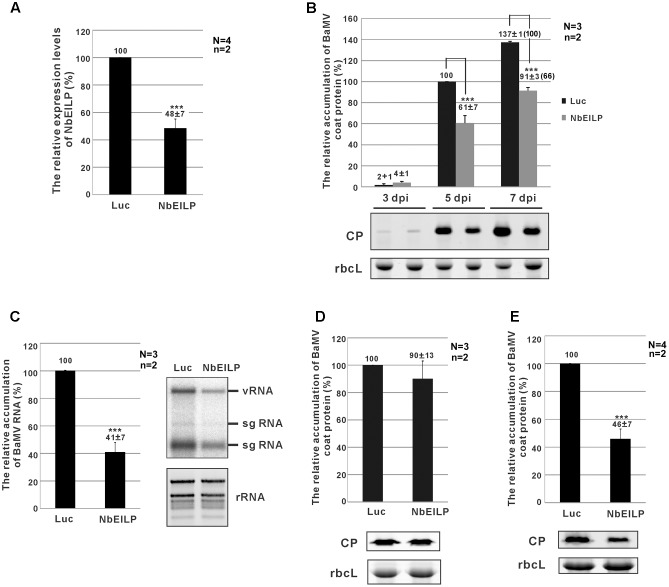
*Bamboo mosaic virus* (BaMV) accumulation in *NbEILP*-knockdown plants. **(A)** The expression level of *NbEILP* was determined by real-time quantitative RT-PCR in *Luc*- and *NbEILP*-knockdown leaves. The numbers above each bar are the mean expression of *NbEILP* with the standard error obtained from at least three independent experiments. **(B)** Western blot analysis of the relative accumulation of BaMV coat protein in *Luc*- and *NbEILP*-knockdown leaves at 3, 5, and 7 days post-inoculation (dpi). The accumulation of BaMV coat protein in *Luc*-knockdown leaves at 5 dpi was set as 100%. The number in the parentheses at 7 dpi is the relative ratio of the accumulation. The representative western blot is shown under the statistical analysis. **(C)** Northern blot analysis of the relative accumulation of BaMV RNA in *Luc*- and *NbEILP*-knockdown leaves in 5 dpi. The accumulation of BaMV genomic RNA in *Luc*-knockdown leaves was set as 100%. The representative Northern blot is shown by the statistical analysis. vRNA: viral genomic RNA; sgRNA: viral subgenomic RNA; rRNA: ribosomal RNAs. **(D,E)** Western blot analysis of the relative accumulation of BaMV coat protein in protoplasts at 24 hpi and systemic leaves at 7 dpi of Luc- and NbEILP-knockdown plants, respectively. The accumulation of BaMV coat protein in *Luc*-knockdown was set as 100%. The numbers above each bar were the relative mean accumulation of BaMV with the standard error derived from the number of independent experimental repeats (N) and the number of plants (n) used in each repeat. Luc: *luciferase*-knockdown plants; NbEILP: *NbEILP*-knockdown plants; CP: coat protein; and rbcL: RuBisCo large subunit used as a loading control. Asterisks indicate statistically significant differences compared with the group indicated (^∗^*P* < 0.05, ^∗∗^*P* < 0.005, ^∗∗∗^*p* < 0.001) by Student’s *t*-test.

To study the relation between *NbEILP* and BaMV accumulation in *N. benthamiana*, TRV-based VIGS was used to knock down *NbEILP* expression before BaMV inoculation. The morphology of *NbEILP*-knockdown plants and control plants (agroinfiltrated with TRV2/Luc) did not differ (Supplementary Figure S3). Real-time qRT-PCR revealed the expression of *NbEILP* reduced to 48% of the wildtype level in knockdown plants (**Figure 2A**). BaMV CP level was significantly reduced to approximately 61 and 66% that of control plants at 5 and 7 dpi (**Figure 2B**). BaMV genomic RNA level was also reduced to approximately 41% in knockdown plants at 5 dpi (**Figure 2C**). However, this effect did not occur on infection with *Potato virus X* and *Cucumber mosaic virus* in *NbEILP*-knockdown plants (Supplementary Figure S4). These results suggest a specific involvement of *NbEILP* in BaMV accumulation.

### NbEILP Is Involved in BaMV Cell-to-Cell Movement

The reduced BaMV accumulation in *NbEILP*-knockdown plants could result from an effect on viral RNA replication or cell-to-cell movement. To reveal which effect is the major cause restricting BaMV accumulation in knockdown plants, we prepared protoplasts from knockdown plants whereby the cell wall was removed and plasmodesmata were excluded. The accumulation of BaMV CP did not differ between knockdown and control protoplasts at 24 h post-inoculation (hpi; **Figure 2D**). Therefore, reduced expression of *NbEILP* had no effect on BaMV replication. Hence, NbEILP may be involved in movement of BaMV in plants.

To determine whether NbEILP also plays a role in long-distance movement, we examined BaMV accumulation in upper, non-inoculated leaves. The accumulation of BaMV CP in *NbEILP*-knockdown plants was reduced to approximately 46% that of the control plants at 7 dpi (**Figure 2E**). Because BaMV accumulation was shown no obvious difference in upper non-inoculated and inoculated leaves (**Figure 2B**), NbEILP has less effect on systemic movement but mainly on cell-to-cell movement.

**FIGURE 3:**
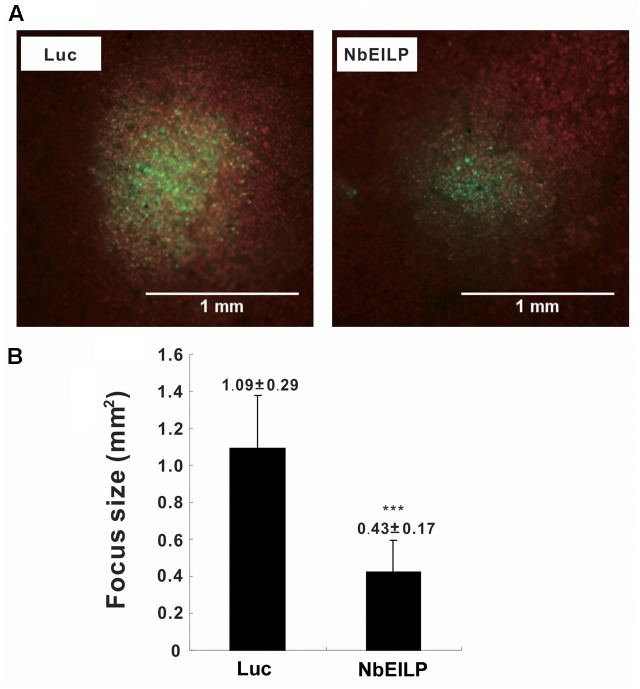
Mean size of BaMV foci on *Luciferase*- and *NbEILP*-knockdown *N. benthamiana* leaves. **(A)** Fluorescent microscopy of GFP foci on inoculated leaves of control (*Luciferase*; Luc) and *NbEILP*-knockdown (NbEILP) plants after inoculation with the infectious plasmid pCBG. Bar length = 1.0 mm and **(B)** GFP focus size (mm^2^). The mean size of foci was derived from 474 lesions (*NbEILP*-knockdown with 184 lesions and control with 290 lesions). Asterisks indicate statistically significant differences compared with the group indicated (^∗∗∗^*p* < 0.001) by Student’s *t*-test.

To validate this conclusion, we examined viral movement in *NbEILP*-knockdown plants. We inoculated knockdown plants with the infectious cDNA plasmid pCBG (Lin et al., 2004), which carries the GFP reporter gene. The number and size of fluorescent foci were examined by fluorescent microscopy. The mean size of BaMV foci was approximately 0.43 mm^2^ in *NbEILP*-knockdown plants and was much smaller than the 1.09 mm^2^ size in control plants (**Figure 3**). Hence, the reduced expression of *NbEILP* restricted BaMV movement in *N. benthamiana* plants.

### NbEILP Is Localized at the ER Membrane

**FIGURE 4:**
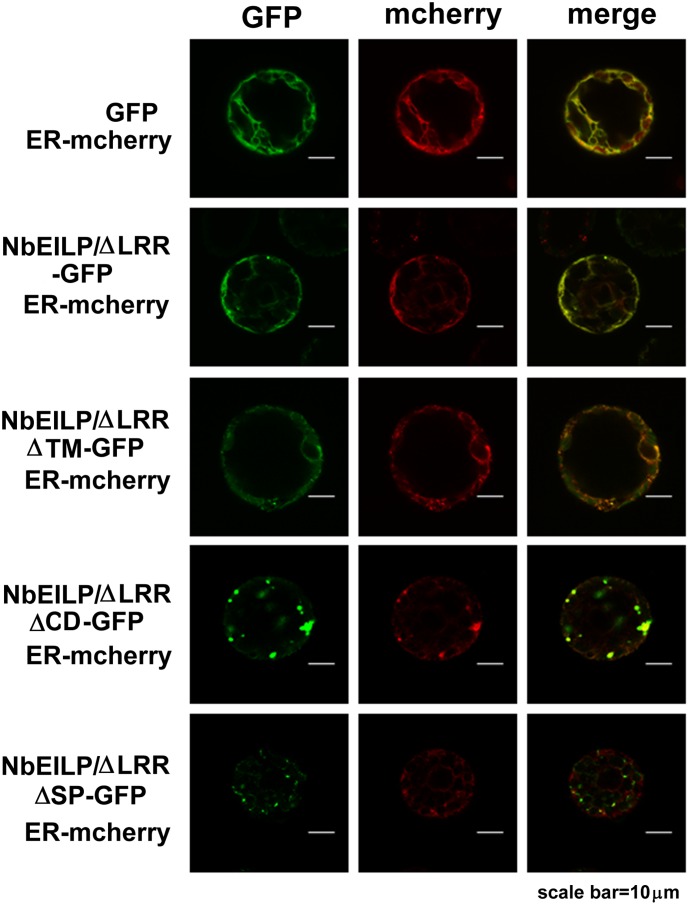
Visualization of NbEILP derivative-GFP and ER–mCherry (ER membrane) constructs in *N. benthamiana* protoplasts. NbEILP derivative constructs were transiently expressed in *N. benthamiana* leaves by agro-infiltration. Confocal microscopy of protoplasts isolated from inoculated leaves. Scale bar: 10 μm.

Because the sequence of NbEILP contains a signal peptide and a transmembrane domain, we would expect NbEILP to be localized at the ER membrane. Therefore, we fused NbEILP and its derivatives with GFP at their C-termini to localize their expression in cells. After transient expression of the full-length NbEILP-GFP, we did not observe any GFP signal in plants. Therefore, we constructed and expressed a short version of NbEILP with deletion of LRR. NbEILP/ΔLRR-GFP and the ER marker (mCherry) co-localized in cells on confocal microscopy. This result confirmed that NbEILP/ΔLRR is localized at the ER membrane (**Figure 4**).

### Expression of NbEILP and NbEILP/ΔLRR Enhance BaMV Accumulation

**FIGURE 5:**
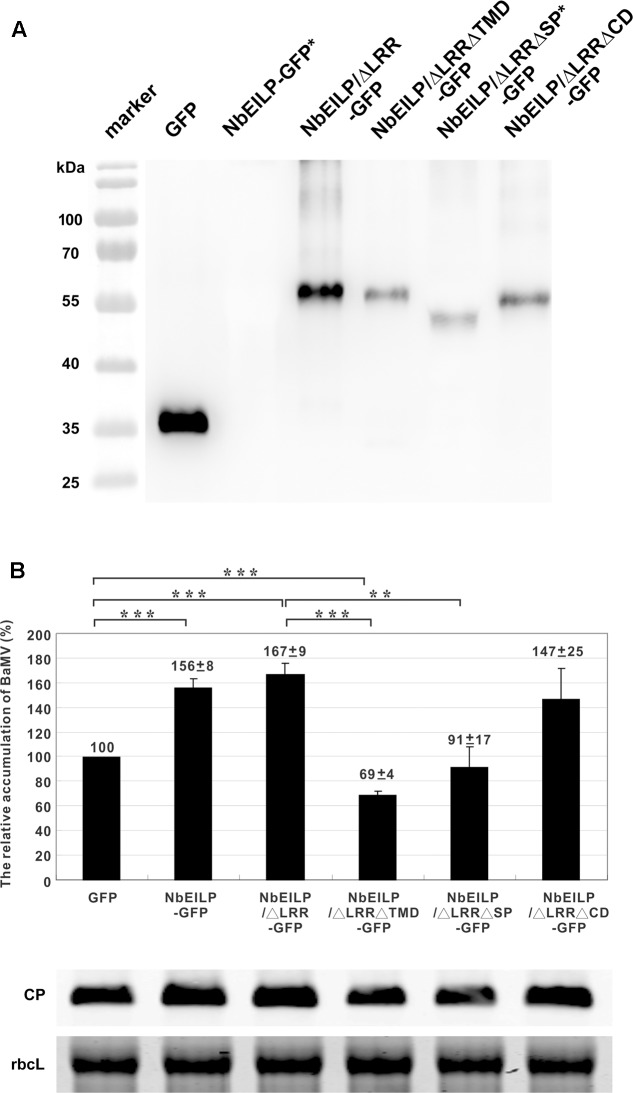
Relative accumulation of BaMV CP in *NbEILP*- and derivative-expressed *N. benthamiana* plants. **(A)** GFP (pBI-mGFP1 vector only) and all NbEILP derivatives were co-infiltrated with p19 silencing suppressor in a 1:1 ratio. After 2 days, total protein was collected and extracted from 100 mg of each infiltrated leaf and detected by western blot analysis. NbEILP-GFP and NbEILP/ΔLRRΔSP-GFP indicated as ^∗^ were 10-fold loaded as compared with other samples. **(B)** Western blot analysis of the relative accumulation of BaMV CP at 4 dpi on *N. benthamiana* leaves with transient expression of GFP, NbEILP-GFP, NbEILP/ΔLRR-GFP, NbEILP/ΔLRRΔTMD-GFP, NbEILP/ΔLRRΔSP-GFP, and NbEILP/ΔLRRΔCD-GFP by agro-infiltration. The data from western blot analysis were normalized to that of the large subunit of RuBisCO (rbcL) as the loading control stained with Coomassie blue. The numbers above bars are the mean and standard error of at least three independent experiments. Asterisks indicate statistically significant differences compared with the group indicated (^∗∗^*P* < 0.01, ^∗∗∗^*p* < 0.001) by Student’s *t*-test.

The results from knockdown experiments suggested that NbEILP could specifically help with BaMV accumulation. To confirm this finding, we transiently expressed NbEILP in *N. benthamiana* plants, but the expression of full-length NbEILP-GFP was not detected in plants even with 10-fold protein loaded (**Figure 5A**). However, the accumulation of BaMV was approximately 1.6-fold that of the control (**Figure 5B**) even though NbEILP expression was under detectable limit on western blot analysis. The expression of NbEILP/ΔLRR-GFP could also enhance the accumulation of BaMV approximately 1.7-fold (**Figure 5B**). Hence, the LRR (790 amino acids), the major portion of NbEILP (**Figure 1**), is not the critical determinant assisting BaMV movement.

### ER Membrane-Association Is Required for NbEILP Assisting BaMV Movement

To determine which domain in NbEILP is involved in BaMV movement, we constructed mutants based on NbEILP/ΔLRR-GFP. The three deletion mutants involved removal of the transmembrane domain (NbEILP/ΔLRRΔTMD-GFP), cyto-plasmic domain (NbEILP/ΔLRRΔCD-GFP), and signal peptide (NbEILP/ΔLRRΔSP-GFP). After proteins were transiently expressed in *N. benthamiana* plants, total protein extracted from leaves was probed with anti-GFP antibody. The expression of NbEILP/ΔLRRΔSP-GFP was limited and could be detected as compared with other proteins when 10-fold more protein was loaded (**Figure 5A**).

*Bamboo mosaic virus* accumulation in the presence of NbEILP/ΔLRRΔTMD-GFP, NbEILP/ΔLRRΔSP-GFP, and NbEILP/ΔLRRΔCD-GFP was 69, 91, and 147%, respectively, that of the control (**Figure 5B**). These results suggest that the transmembrane domain is the most critical region involved on BaMV accumulation. Mutant NbEILP/ΔLRRΔTMD-GFP retaining the signal peptide but no transmembrane domain presumably could pass through the ER membrane into the lumen. With this process, NbEILP/ΔLRRΔTMD-GFP has a dominant-negative effect on BaMV accumulation (reduced to 69% compared to the control). The signal peptide is also critical in that it directs NbEILP targeting to the ER membrane. As compared with NbEILP/ΔLRR-GFP, with NbEILP/ΔLRRΔSP-GFP the accumulation of BaMV CP in leaves was significantly reduced from 167 to 91% (**Figure 5B**), with no significant difference from that of the control GFP alone. Thus, NbEILP/ΔLRRΔSP-GFP with removal of the signal peptide perhaps could not target the ER failing to assist BaMV movement. The cytoplasmic domain is irrelevant for BaMV accumulation because NbEILP/ΔLRRΔCD-GFP containing the signal peptide and transmembrane domain could still help with BaMV movement.

These results indicated that the variants of EILP proteins that are associated with the ER membrane upon expression by these constructs, such as NbEILP, NbEILP/ΔLRR-GFP, and NbEILP/ΔLRRΔCD-GFP are involved in BaMV accumulation. Confocal images of NbEILP/ΔLRR-GFP and NbEILP/ΔLRRΔCD-GFP confirmed this suggestion (**Figure 4**). Mutant NbEILP/ΔLRRΔSP-GFP without the signal peptide did not co-localize with the ER marker (**Figure 4**) and failed to assist in BaMV movement. However, NbEILP/ΔLRRΔTMD-GFP co-localized with the ER but somehow had weaker signals than the constructs containing the transmembrane domain. The mutant NbEILP/ΔLRRΔTMD-GFP, containing the signal peptide but not the transmembrane domain, could be directed to enter the ER lumen.

### NbEILP/ΔLRR-GFP and NbEILP/ΔLRRΔCD-GFP Are Membrane-Associated

**FIGURE 6:**
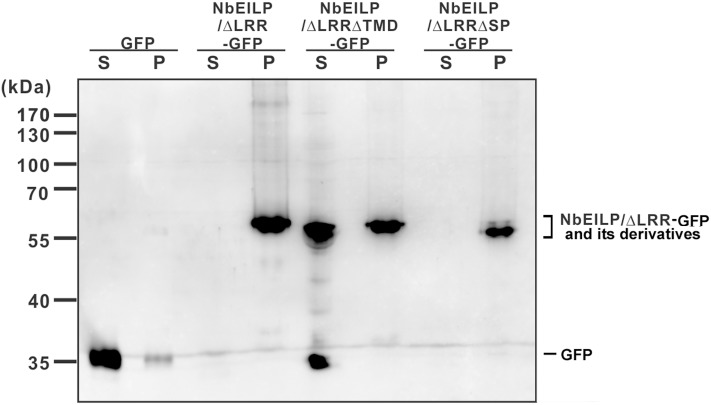
Western blot analysis of transiently expressed proteins. Total protein was extracted from *N. benthamiana* plants with transient expression of GFP or NbEILP/ΔLRR-GFP and its derivatives. The extracted proteins were fractionated into soluble (S) and membrane-associated (P) fractions, and separated on a 12% SDS-polyacrylamide gel for western blot analysis.

To validate the confocal microscopy findings, we fractionated the total protein extracted from BaMV-infiltrated leaves. NbEILP/ΔLRR-GFP and NbEILP/ΔLRRΔCD-GFP were membrane-associated, whereas NbEILP/ΔLRRΔTMD-GFP, retaining the signal peptide but not transmembrane domain, was mostly distributed to the soluble fraction (**Figure 6**).

Results from localization and BaMV accumulation experiments suggest that NbEILP and its derivatives localizing at the ER membrane could enhance BaMV accumulation. The results also imply that NbEILP localizing at the ER membrane plays a critical role in assisting BaMV movement.

## Discussion

In this study, we cloned a gene upregulated after BaMV infection in *N. benthamiana* and determined how it is involved in virus accumulation. The full-length cDNA of this gene is approximately 3.2 kb. The sequence for the polypeptide (NbEILP) encoded from this gene is similar to that of *Cf-2/Cf-5* genes in tomato, involved in resistance to strains of *C. fulvum* (Dixon et al., 1996), and the EILP in tobacco induced by treatment with salicylic acid (Takemoto et al., 2000). NbEILP has 32 LRRs, a transmembrane domain, and a short cytoplasmic domain. The LRR-containing domains are present in more than 60,000 proteins that are widespread in viruses, bacteria, archaea, and eukaryotes (Kobe and Kajava, 2001; Bella et al., 2008). All of these proteins have 20 to 30 residues in length in the conserved region LxxLxLxxNxL (L: Leu, Ile, Val, or Phe; N: Asn, Thr, Ser, or Cys; and x is any amino acid), which forms a highly conserved repeated β-strand/β-turn structural motif in LRR proteins (Kobe and Deisenhofer, 1994). LRR domains are known as sites of protein–protein interaction, peptide–ligand binding, and protein–carbohydrate interaction that play a significant role in plant pathogen resistance (Jones and Jones, 1997; Kajava, 1998).

Although NbEILP shows sequence similarity to the resistance gene, it is specifically involved in BaMV movement. We did not observe any effect on the accumulation of PVX and CMV in *NbEILP*-knockdown plants (Supplementary Figure S4). Because NbEILP is a large protein (969 amino acids), transient expression of this protein *in planta* is difficult (**Figure 5A**). However, even with limited expression (undetectable on western blot analysis), the protein still could assist with BaMV accumulation (**Figure 5B**). The expression of NbEILP/ΔLRR-GFP, with removal of LRR, could still retain the ability to assist BaMV movement. The LRR domain in NbEILP may not play an essential role in BaMV movement. However, the expression level of NbEILP is at least 10-fold lower than that of NbEILP/ΔLRR; they can assist BaMV accumulation to a similar level (**Figure 5**). Therefore, we still could not rule out the possibility of the involvement of LRR in BaMV movement.

The results with the NbEILP derivatives indicated that only the constructs targeting the ER and retained on the membrane were involved in BaMV movement. The results imply that NbEILP could interact with the viral-encoded movement complex or gather the host-encoded factors that are crucial for viral movement. Therefore, NbEILP/ΔLRRΔTMD-GFP localized in the lumen of the ER (most likely), revealed to have a dominant-negative effect on BaMV accumulation (**Figure 5B**), could result from the viral movement complex, or the host factor crucial for the movement was trapped in the lumen.

For successful and efficient movement of a viral pathogen, different host factors were used to target the viral RNA replication particles and/or TGB proteins (in potexviruses) to the plasmodesmata via the endomembrane secretory pathway (most likely the ER and post-ER secretory pathway) (Ju et al., 2005; Tseng et al., 2009; Yoshimoto et al., 2010; Tilsner et al., 2012). A common strategy used by most of the positive-sense single-stranded RNA virus is to use the endomembrane systems for viral RNA replication (Pena and Heinlein, 2012; Tilsner et al., 2012). The secretory pathway is also commonly used for the newly synthesized progeny RNAs moving from the replication site to neighboring cells. TGBp1 of BaMV interacts with the viral RNA (Wung et al., 1999; Liou et al., 2000; Lin et al., 2004). TGBp2 and TGBp3 of BaMV are the ER-associated transmembrane proteins (Hsu et al., 2008; Tseng et al., 2009; Chou et al., 2013). As a native pathway of NbEILP for sensing the elicitors from pathogens, NbEILP is synthesized and retained on the ER membrane and is possibly secreted to the plasma membrane as vesicle trafficking. The movement complex containing TGBp1, vRNA, replicase, CP, TGBp2, and TGBp3 of BaMV (Chou et al., 2013; Huang et al., 2017) might take advantage of the secretory path of NbEILP to reach the plasmodesmata for cell-to-cell movement. Therefore, the possible role of NbEILP in BaMV movement could be gathering these viral- or host-encoded movement-associated proteins together. Here we identified a host protein NbEILP from *N. benthamiana* that is involved in BaMV cell-to-cell movement.

## Author Contributions

I-HC and C-HsT designed the research, analyzed the data and wrote the manuscript. Y-PH, C-HTse, J-TN, and C-HTsa performed the research. Y-HH and C-HsT participated in data analysis and discussion.

## Conflict of Interest Statement

The authors declare that the research was conducted in the absence of any commercial or financial relationships that could be construed as a potential conflict of interest.

## Footnotes

1 http://rsbweb.nih.gov/ij/

## References

[B1] Ahlquist P. Noueiry A. O. Lee W. M. Kushner D. B. Dye B. T. (2003). Host factors in positive-strand RNA virus genome replication. *J. Virol.* 77 8181–8186. 10.1128/JVI.77.15.8181-8186.2003 12857886 PMC165243

[B2] Bella J. Hindle K. L. Mcewan P. A. Lovell S. C. (2008). The leucine-rich repeat structure. *Cell Mol. Life. Sci.* 65 2307–2333. 10.1007/s00018-008-8019-0 18408889 PMC11131621

[B3] Chen I. H. Chou W. J. Lee P. Y. Hsu Y. H. Tsai C. H. (2005). The AAUAAA motif of *Bamboo mosaic virus* RNA is involved in minus-strand RNA synthesis and plus-strand RNA polyadenylation. *J. Virol.* 79 14555–14561. 10.1128/JVI.79.23.14555-14561.2005 16282455 PMC1287560

[B4] Chen I. H. Huang Y. W. Tsai C. H. (2017). The functional roles of the *cis*-acting elements in *Bamboo mosaic virus* RNA genome. *Front. Microbiol.* 8:645 10.3389/fmicb.2017.00645 PMC5390519 28450857

[B5] Cheng S. F. Huang Y. P. Wu Z. R. Hu C. C. Hsu Y. H. Tsai C. H. (2010). Identification of differentially expressed genes induced by *Bamboo mosaic virus* infection in *Nicotiana benthamiana* by cDNA-amplified fragment length polymorphism. *BMC Plant Biol.* 10:286 10.1186/1471-2229-10-286 PMC3024324 21184690

[B6] Chou Y. L. Hung Y. J. Tseng Y. H. Hsu H. T. Yang J. Y. Wung C. H. (2013). The stable association of virion with the triple-gene-block protein 3-based complex of *Bamboo mosaic virus*. *PLOS Pathog.* 9:e1003405 10.1371/journal.ppat.1003405 PMC3675025 23754943

[B7] Dangl J. L. Jones J. D. (2001). Plant pathogens and integrated defence responses to infection. *Nature* 411 826–833. 10.1038/35081161 11459065

[B8] de Wit P. J. Joosten M. H. (1999). Avirulence and resistance genes in the *Cladosporium fulvum*-tomato interaction. *Curr. Opin. Microbiol.* 2 368–373. 10.1016/S1369-5274(99)80065-4 10458978

[B9] DiMaio F. Chen C. C. Yu X. Frenz B. Hsu Y. H. Lin N. S. (2015). The molecular basis for flexibility in the flexible filamentous plant viruses. *Nat. Struct. Mol. Biol.* 22 642–644. 10.1038/nsmb.3054 26167882 PMC4527879

[B10] Dixon M. S. Golstein C. Thomas C. M. Van Der Biezen E. A. Jones J. D. (2000). Genetic complexity of pathogen perception by plants: the example of Rcr3, a tomato gene required specifically by Cf-2. *Proc. Natl. Acad. Sci. U.S.A.* 97 8807–8814. 10.1073/pnas.97.16.8807 10922039 PMC34016

[B11] Dixon M. S. Jones D. A. Keddie J. S. Thomas C. M. Harrison K. Jones J. D. (1996). The tomato Cf-2 disease resistance locus comprises two functional genes encoding leucine-rich repeat proteins. *Cell* 84 451–459. 10.1016/S0092-8674(00)81290-8 8608599

[B12] Ho T. L. Lee H. C. Chou Y. L. Tseng Y. H. Huang W. C. Wung C. H. (2017). The cysteine residues at the C-terminal tail of *Bamboo mosaic virus* triple gene block protein 2 are critical for efficient plasmodesmata localization of protein 1 in the same block. *Virology* 501 47–53. 10.1016/j.virol.2016.11.005 27863274

[B13] Howard A. R. Heppler M. L. Ju H. J. Krishnamurthy K. Payton M. E. Verchot-Lubicz J. (2004). *Potato virus X* TGBp1 induces plasmodesmata gating and moves between cells in several host species whereas CP moves only in *N. benthamiana* leaves. *Virology* 328 185–197. 10.1016/j.virol.2004.06.039 15464839

[B14] Hsu H. T. Chou Y. L. Tseng Y. H. Lin Y. H. Lin T. M. Lin N. S. (2008). Topological properties of the triple gene block protein 2 of *Bamboo mosaic virus*. *Virology* 379 1–9. 10.1016/j.virol.2008.06.019 18639913

[B15] Huang C. Y. Tsai C. H. (1998). Evolution of *Bamboo mosaic virus* in a nonsystemic host results in mutations in the helicase-like domain that cause reduced RNA accumulation. *Virus Res.* 58 127–136. 10.1016/S0168-1702(98)00109-9 9879769

[B16] Huang Y. L. Hsu Y. H. Han Y. T. Meng M. (2005). mRNA guanylation catalyzed by the S-adenosylmethionine-dependent guanylyltransferase of *Bamboo mosaic virus*. *J. Biol. Chem.* 280 13153–13162. 10.1074/jbc.M412619200 15677480

[B17] Huang Y. P. Chen I. H. Tsai C. H. (2017). Host factors in the infection cycle of *Bamboo mosaic virus*. *Front. Microbiol.* 8:437 10.3389/fmicb.2017.00437 PMC5350103 28360904

[B18] Hyodo K. Okuno T. (2016). Pathogenesis mediated by proviral host factors involved in translation and replication of plant positive-strand RNA viruses. *Curr. Opin. Virol.* 17 11–18. 10.1016/j.coviro.2015.11.004 26651023

[B19] Jones D. A. Jones J. D. G. (1997). The role of leucine-rich repeat proteins in plant defences. *Adv. Bot. Res.* 24 89–167. 10.1016/S0065-2296(08)60072-5

[B20] Joosten M. de Wit P. (1999). The tomato-*Cladosporium fulvum* interaction: a versatile experimental system to study plant-pathogen interactions. *Annu. Rev. Phytopathol.* 37 335–367. 10.1146/annurev.phyto.37.1.335 11701827

[B21] Ju H. J. Samuels T. D. Wang Y. S. Blancaflor E. Payton M. Mitra R. (2005). The *Potato virus X* TGBp2 movement protein associates with endoplasmic reticulum-derived vesicles during virus infection. *Plant Physiol.* 138 1877–1895. 10.1104/pp.105.066019 16055678 PMC1183379

[B22] Kajava A. V. (1998). Structural diversity of leucine-rich repeat proteins. *J. Mol. Biol.* 277 519–527. 10.1006/jmbi.1998.1643 9533877

[B23] Kobe B. Deisenhofer J. (1994). The leucine-rich repeat: a versatile binding motif. *Trends Biochem. Sci.* 19 415–421. 10.1016/0968-0004(94)90090-6 7817399

[B24] Kobe B. Kajava A. V. (2001). The leucine-rich repeat as a protein recognition motif. *Curr. Opin. Struct. Biol* 11 725–732. 10.1016/S0959-440X(01)00266-4 11751054

[B25] Krishnamurthy K. Heppler M. Mitra R. Blancaflor E. Payton M. Nelson R. S. (2003). The *Potato virus X* TGBp3 protein associates with the ER network for virus cell-to-cell movement. *Virology* 309 135–151. 10.1016/S0042-6822(02)00102-2 12726734

[B26] Laliberte J. F. Beauchemin C. Boutet N. (2007). Visualization of the interaction between the precursors of VPg, the viral protein linked to the genome of *Turnip mosaic virus*, and the translation eukaryotic initiation factor iso 4E in planta. *J. Virol.* 81 775–782. 10.1128/JVI.01277-06 17079311 PMC1797466

[B27] Lan P. Yeh W. B. Tsai C. W. Lin N. S. (2010). A unique glycine-rich motif at the N-terminal region of *Bamboo mosaic virus* coat protein is required for symptom expression. *Mol. Plant Microbe Interact.* 23 903–914. 10.1094/MPMI-23-7-0903 20521953

[B28] Lee C. C. Ho Y. N. Hu R. H. Yen Y. T. Wang Z. C. Lee Y. C. (2011). The interaction between *Bamboo mosaic virus* replication protein and coat protein is critical for virus movement in plant hosts. *J. Virol.* 85 12022–12031. 10.1128/JVI.05595-11 21917973 PMC3209275

[B29] Li Y. I. Chen Y. J. Hsu Y. H. Meng M. (2001a). Characterization of the AdoMet-dependent guanylyltransferase activity that is associated with the N terminus of *Bamboo mosaic virus* replicase. *J. Virol.* 75 782–788. 11134291 10.1128/JVI.75.2.782-788.2001 PMC113974

[B30] Li Y. I. Cheng Y. M. Huang Y. L. Tsai C. H. Hsu Y. H. Meng M. (1998). Identification and characterization of the *Escherichia coli*-expressed RNA-dependent RNA polymerase of *Bamboo mosaic virus*. *J. Virol.* 72 10093–10099. 9811749 10.1128/jvi.72.12.10093-10099.1998 PMC110542

[B31] Li Y. I. Shih T. W. Hsu Y. H. Han Y. T. Huang Y. L. Meng M. (2001b). The helicase-like domain of plant potexvirus replicase participates in formation of RNA 5′ cap structure by exhibiting RNA 5′-triphosphatase activity. *J. Virol.* 75 12114–12120. 11711602 10.1128/JVI.75.24.12114-12120.2001 PMC116107

[B32] Lin M. K. Chang B. Y. Liao J. T. Lin N. S. Hsu Y. H. (2004). Arg-16 and Arg-21 in the N-terminal region of the triple-gene-block protein 1 of *Bamboo mosaic virus* are essential for virus movement. *J. Gen. Virol.* 85 251–259. 10.1099/vir.0.19442-0 14718640

[B33] Lin M. K. Hu C. C. Lin N. S. Chang B. Y. Hsu Y. H. (2006). Movement of potexviruses requires species-specific interactions among the cognate triple gene block proteins, as revealed by a trans-complementation assay based on the *Bamboo mosaic virus* satellite RNA-mediated expression system. *J. Gen. Virol.* 87 1357–1367. 10.1099/vir.0.81625-0 16603539

[B34] Lin M. T. Kitajima W. E. Cupertino P. F. Costa C. L. (1977). Partial purification and some properties of *Bamboo mosaic virus*. *Phytopath* 67 1439–1443. 10.1094/Phyto-67-1439

[B35] Lin N. S. Lin B. Y. Lo N. W. Hu C. C. Chow T. Y. Hsu Y. H. (1994). Nucleotide sequence of the genomic RNA of *Bamboo mosaic potexvirus*. *J. Gen. Virol.* 75 2513–2518. 10.1099/0022-1317-75-9-2513 8077956

[B36] Liou D. Y. Hsu Y. H. Wung C. H. Wang W. H. Lin N. S. Chang B. Y. (2000). Functional analyses and identification of two arginine residues essential to the ATP-utilizing activity of the triple gene block protein 1 of *Bamboo mosaic potexvirus*. *Virology* 277 336–344. 10.1006/viro.2000.0610 11080481

[B37] Meng M. Lee C. C. (2017). Function and structural organization of the replication protein of *Bamboo mosaic virus*. *Front. Microbiol.* 8:522 10.3389/fmicb.2017.00522 PMC5368238 28400766

[B38] Mitra R. Krishnamurthy K. Blancaflor E. Payton M. Nelson R. S. Verchot-Lubicz J. (2003). The *Potato virus X* TGBp2 protein association with the endoplasmic reticulum plays a role in but is not sufficient for viral cell-to-cell movement. *Virology* 312 35–48. 10.1016/S0042-6822(03)00180-6 12890619

[B39] Nagy P. D. (2016). Tombusvirus-host interactions: co-opted evolutionarily conserved host factors take center court. *Annu. Rev. Virol.* 3 491–515. 10.1146/annurev-virology-110615-042312 27578441

[B40] Nelson B. K. Cai X. Nebenfuhr A. (2007). A multicolored set of in vivo organelle markers for co-localization studies in Arabidopsis and other plants. *Plant J.* 51 1126–1136. 10.1111/j.1365-313X.2007.03212.x 17666025

[B41] Park M. R. Jeong R. D. Kim K. H. (2014). Understanding the intracellular trafficking and intercellular transport of potexviruses in their host plants. *Front. Plant Sci.* 5:60 10.3389/fpls.2014.00060 PMC3957223 24672528

[B42] Pena E. J. Heinlein M. (2012). RNA transport during TMV cell-to-cell movement. *Front. Plant Sci.* 3:193 10.3389/fpls.2012.00193 PMC3428586 22973280

[B43] Perbal M. C. Thomas C. L. Maule A. J. (1993). Cauliflower mosaic virus gene I product (P1) forms tubular structures which extend from the surface of infected protoplasts. *Virology* 195 281–285. 10.1006/viro.1993.1375 8317106

[B44] Ruiz M. T. Voinnet O. Baulcombe D. C. (1998). Initiation and maintenance of virus-induced gene silencing. *Plant Cell* 10 937–946. 10.1105/tpc.10.6.937 9634582 PMC144041

[B45] Ryabov E. V. Robinson D. J. Taliansky M. E. (1999). A plant virus-encoded protein facilitates long-distance movement of heterologous viral RNA. *Proc. Natl. Acad. Sci. U.S.A.* 96 1212–1217. 10.1073/pnas.96.4.1212 9990003 PMC15442

[B46] Shaner N. C. Campbell R. E. Steinbach P. A. Giepmans B. N. Palmer A. E. Tsien R. Y. (2004). Improved monomeric red, orange and yellow fluorescent proteins derived from *Discosoma* sp. red fluorescent protein. *Nat. Biotechnol.* 22 1567–1572. 10.1038/nbt1037 15558047

[B47] Takemoto D. Hayashi M. Doke N. Mishimura M. Kawakita K. (2000). Isolation of the gene for EILP, an elicitor-inducible LRR receptor-like protein, from tobacco by differential display. *Plant Cell Physiol.* 41 458–464. 10.1093/pcp/41.4.458 10845459

[B48] Tilsner J. Linnik O. Wright K. M. Bell K. Roberts A. G. Lacomme C. (2012). The TGB1 movement protein of *Potato virus X* reorganizes actin and endomembranes into the X-body, a viral replication factory. *Plant Physiol.* 158 1359–1370. 10.1104/pp.111.189605 22253256 PMC3291258

[B49] Tsai C.-H. Cheng C.-P. Peng C.-W. Lin B.-Y. Lin N.-S. Hsu Y.-H. (1999). Sufficient length of a poly(A) tail for the formation of a potential pseudoknot is required for efficient replication of Bamboo mosaic potexvirus RNA. *J. Virol.* 73 2703–2709. 10074116 10.1128/jvi.73.4.2703-2709.1999 PMC104026

[B50] Tsai C. H. Dreher T. W. (1991). *Turnip yellow mosaic virus* RNAs with anticodon loop substitutions that result in decreased valylation fail to replicate efficiently. *J. Virol.* 65 3060–3067. 2033666 10.1128/jvi.65.6.3060-3067.1991 PMC240961

[B51] Tseng Y. H. Hsu H. T. Chou Y. L. Hu C. C. Lin N. S. Hsu Y. H. (2009). The two conserved cysteine residues of the triple gene block protein 2 are critical for both cell-to-cell and systemic movement of *Bamboo mosaic virus*. *Mol. Plant Microbe Interact.* 22 1379–1388. 10.1094/MPMI-22-11-1379 19810807

[B52] Wolf S. Deom C. M. Beachy R. N. Lucas W. J. (1989). Movement protein of *Tobacco mosaic virus* modifies plasmodesmatal size exclusion limit. *Science* 246 377–379. 10.1126/science.246.4928.377 16552920

[B53] Wung C. H. Hsu Y. H. Liou D. Y. Huang W. C. Lin N. S. Chang B. Y. (1999). Identification of the RNA-binding sites of the triple gene block protein 1 of *Bamboo mosaic potexvirus*. *J. Gen. Virol.* 80(Pt 5), 1119–1126. 10.1099/0022-1317-80-5-1119 10355757

[B54] Yoshimoto K. Takano Y. Sakai Y. (2010). Autophagy in plants and phytopathogens. *FEBS Lett.* 584 1350–1358. 10.1016/j.febslet.2010.01.007 20079356

