# Supplementary material for: Nicotiana benthamiana Elicitor-Inducible Leucine-Rich Repeat Receptor-Like Protein Assists Bamboo Mosaic Virus Cell-to-Cell Movement
Source: Front Plant Sci. 2017 Oct 6;8:1736. doi: 10.3389/fpls.2017.01736 (PMC5635722; doi:10.3389/fpls.2017.01736)
Supplement: Supplementary file 1 [file Image_1.PDF]

```
       1                                                                                           93
NbEILP MMMVPKLSSLLQFFTLLYLFAVTFAASTEEATALLKWKATFKNQNNSLLASWTEISSNACRDWYGVECFNGRVSKLDIPDASIIGTLYDFPFS
NtEILP MMMVGKLFSLLHFFTLFCLFTVTFAS-TKEATALLKWKATLQNQSNSLLVSWTPSSK-ACKSWYGVVCFNGRVSKLDIPYAGVIGTLNNFPFS

       94                                                                                         186
NbEILP SLHFLEYVDLSMNNLYGTIPPEIGKLANLVYLDLSLNQISGPIPKEIGYLRSLTMLSLYTNFFSGSIPASLGNLTNLSILYLYRNHLSGPIPD
NtEILP SLPFLEYIDLSMNQLFGSIPPEIGKLTNLVYLDLSFNQISG------------------T------IPPQIGSLAKLQTLHILDNHLNGSIPG

       187                                                                                        279
NbEILP EIGNLRSLTDLELSSNTLDGSIPASLGNLTNLSILYLFNNHLSGPIPEEIGHLVSLTKLALNSNILDGSIPTSLGDSTNLSLLYFFNNHLSGP
NtEILP EIGHLRSLTELDLSINTLNGSIPPSLGNLHNLSLLCLYKNNISGFIPEEIGYLSSLIQLDLNTNFLN--------------------------

       280                                                                                        372
NbEILP IPEEIGHLVSLSDLQLSSNTLDGSIPASLGNLTNLSILYLYNNHLSGPIPVEIGNLRSLTDLELSNNTLDGSIPASLGNLTNLSFLYLNSNHP
NtEILP ----------------------GSIPASLENLHNLSLLYLYENQLSGSIPDEIGQLRTLTDIRLNTNFLTGSIPASLGNLTSLSILQLEHNQL

       373                                                                                        465
NbEILP SGPIPDEIGNLRSLTKLALNSNILDGSIPTSLGNSTNLSLLYLFDNHLSGPIPEEIGHLVSLSDLQLSNNTLDGSIPASLGNLTNLSILYLYS
NtEILP SGSIPEEIGYLRTLAVLSLYTNFLNGSIPISLGNLTSLSSLSLYENHLSG------------------------PIPSSLGNLDNLVYLYLYA

       466                                                                                        558
NbEILP NHLSGPIPNEIGNLRSLVKLELSNNILNGSIPAPLGNLRNLQTLSLAESNLTEEIPSSICNLTLLKYLLLERNNLEGKILQCLSNISGLYVLM
NtEILP NQLSGPIPSELGNLKNLNYMKLHDNQLNGSIPASFGNLRNMQYLFLESNNLTGEIPLSICNLMSLKVLSLGRNSLKGDILQCLINISRLQVLK

       559                                                                                        651
NbEILP MSHNNLSGEIPSSICNLTSLQILDLGRNNMKGEIPQCFGNMSDYLEILDMHGNNLSGTLPITFSTRSVLRSFNLRGNELEGEIPRSLANCKEL
NtEILP IPDNNLSEEIPSSICNLTSLRILDLSRNNLKGSIPQCFGDMGGHLEVLDIHKNGISGTLPTTFRIGSVLRSFTLHENELEGKIPRSLANCKEL

       652                                                                                        744
NbEILP QVLDLGYNHPNDTSPMWLGILSKLQVLSLRSNKLHGPIRTSAIGNLFPKLRILDLSYNAFTDNLPTSLFQHLKAMRTIYRTMETPRYLGEG--
NtEILP QVLDLGDNLLNDTFPMWLGTLPKLQVLRLKSNKLYGSIRTSKDENMFLELRIINLSYNAFTGNIPTSLFQQLKAMRKIDQTVKEPTYLGKFGA

       745                                                                                        837
NbEILP ---YYHDSITVATKGLELELVRILTVYTAIDLSSNRFEGQIPSIMGDLIALRVLNLSHNRLQGRIPPSLGDLTLVESLDLSVNQLVGKIPEQL
NtEILP DIREYNYSVTVTTKGLELKLVRILTVYIIIDLSSNRFEGHVPSIMGELIALRVLNLSRNGLQGHIPPSLGNLFVIESLDLSFNQLSGEIPQQI

       838                                                                                        930
NbEILP ASQITCLEVLNLSYNHLEGCIPQGPQFATFQKNTYEGNDGLRGFPISKGCGNNKVSDTNNTTSALADQGNNSEFLNDFWKAAVMGYGSGLIIG
NtEILP ASQLTSLAVLNLSYNHLQGCIPQGPQFHTFENNSYEGNDGLRGFPISKGCGNDRVSETNNTVSTLDDQESTSEFLNDFWKAALMGYGSGLCIG

       931                                        974
NbEILP LSIAYFMLSARTPNWLSWIVEELEYRITMRRRRKQRACQRHNRR
NtEILP LSILYFMISTGKLKWLSRITEWLQNRFII---------------
```

Figure S1| Amino acid sequence alignment of NbEILP from *Nicotiana benthamiana* and NtEILP (GenBank accession no. BAA88636.1) from *N. tobacum.*

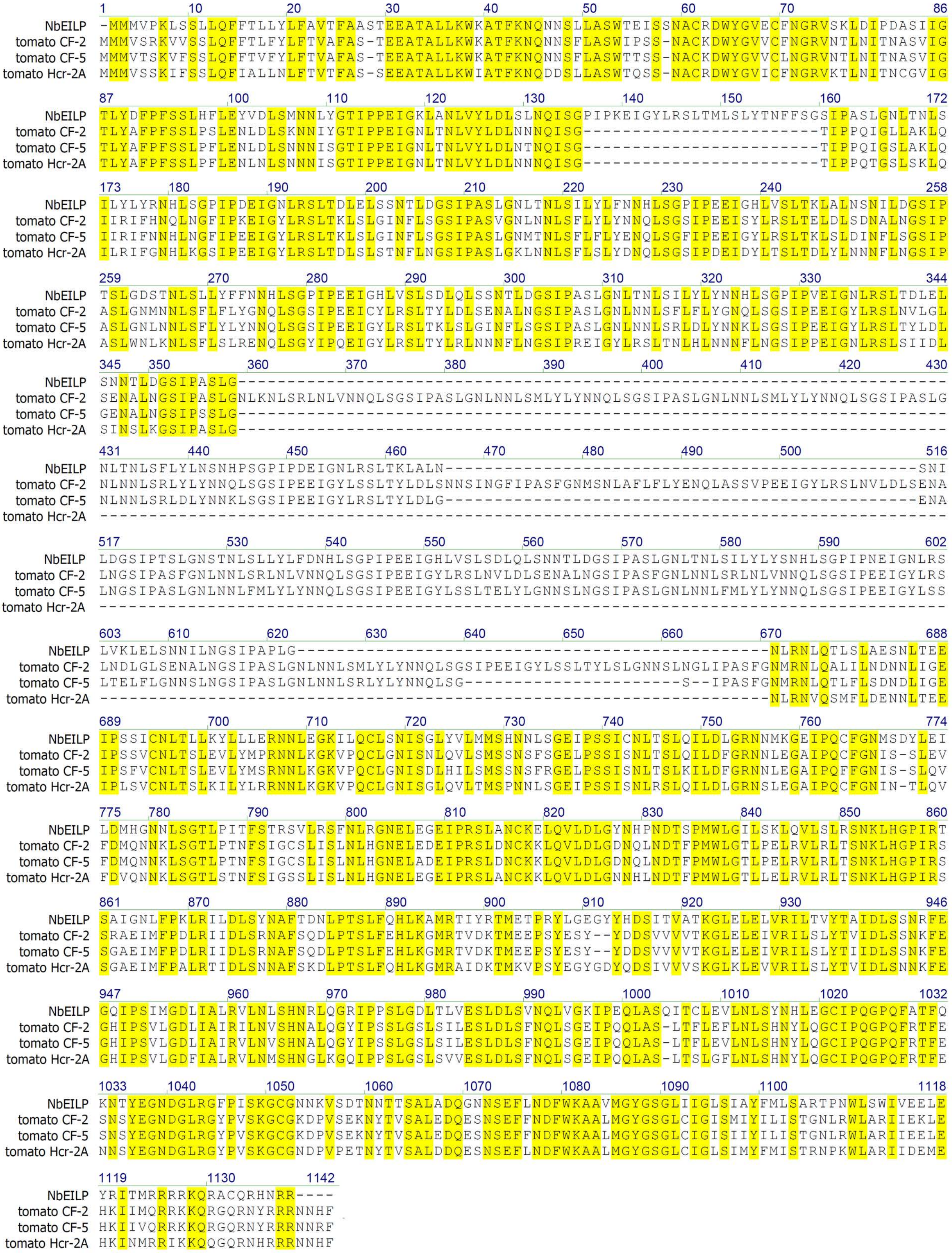

Figure S2| Amino acid sequence alignment of NbEILP from *N. benthamiana* and *CF-2* (GenBank accession no. CAA03427.1), *CF-5* (GenBank accession no. AF053993) and *Hcr2-2A* (GenBank accession no. AAC78594.1) from tomato.

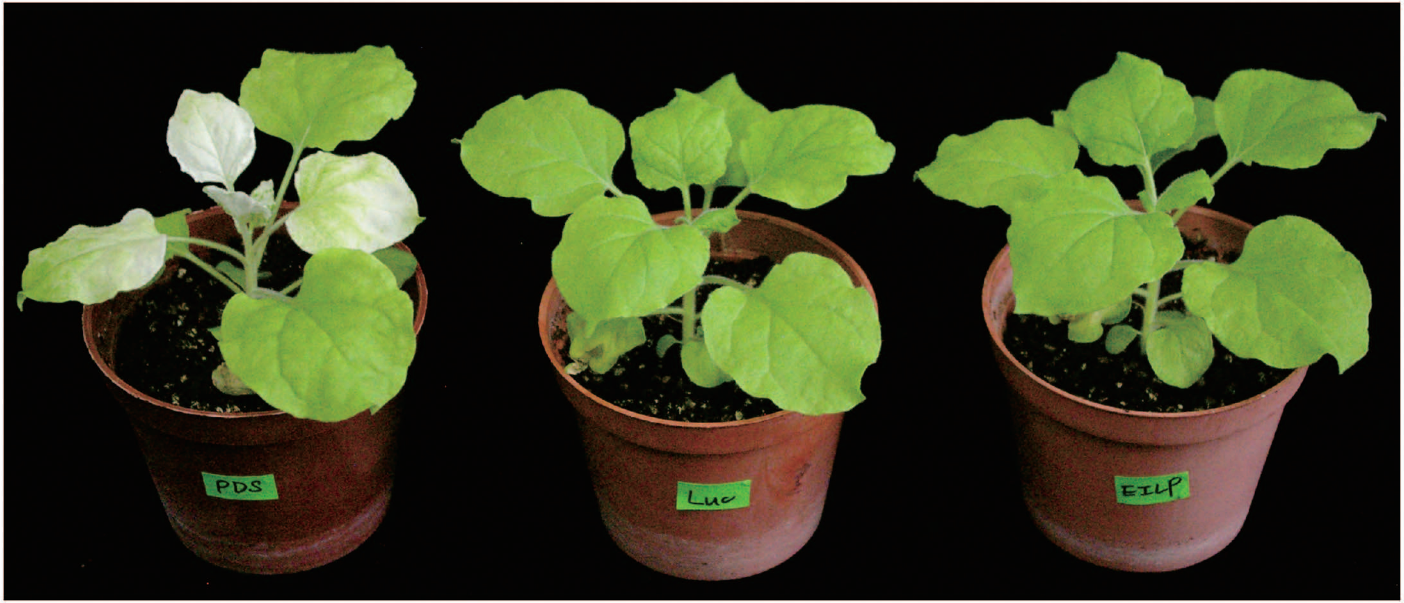

PDS Luc NbEILP

Figure S3| Morphology of Luc- and NbEILP-knockdown plants. Luc (*Luciferase* knockdown) and NbEILP (*NbEILP* knockdown).

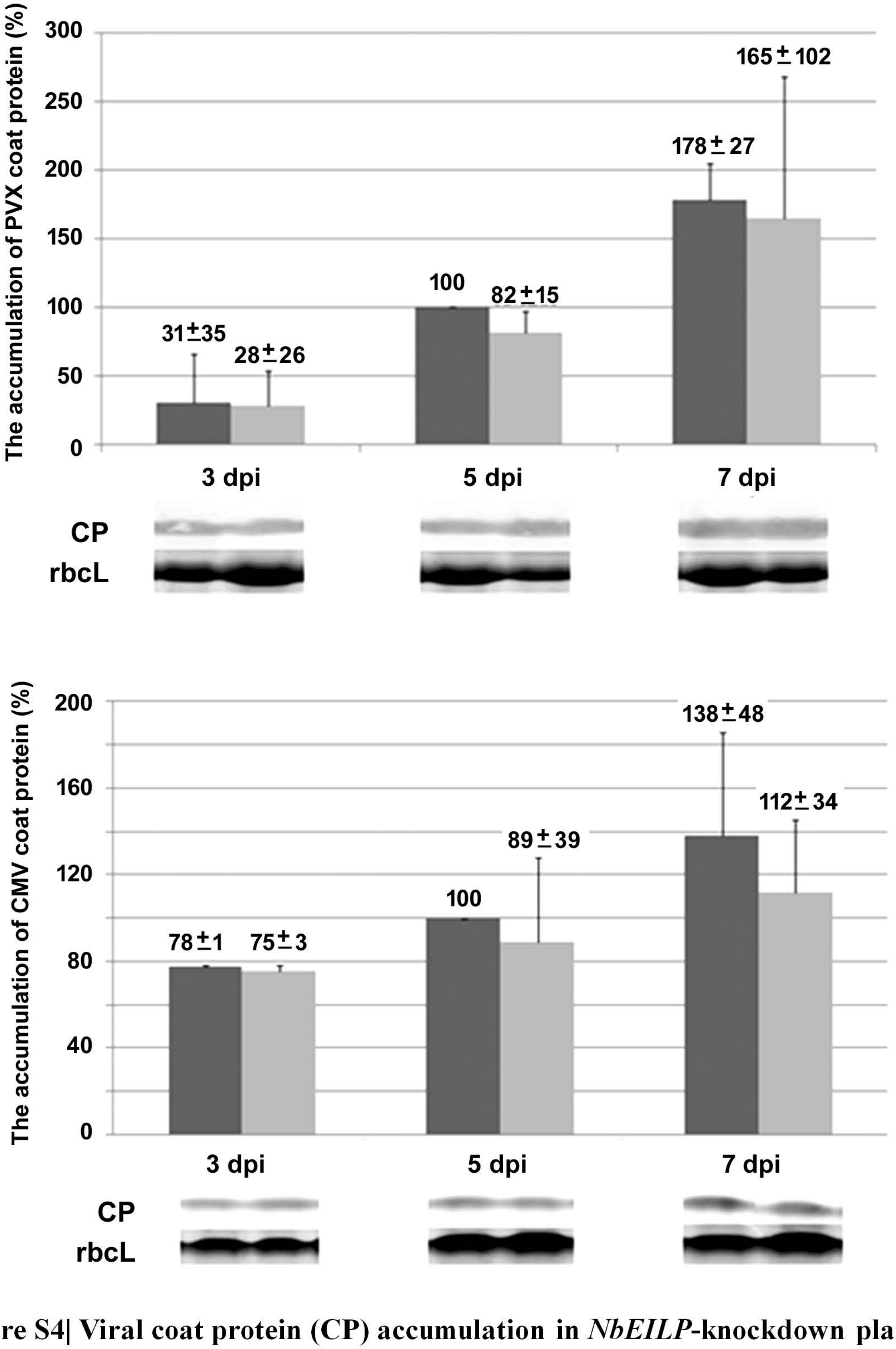

Figure S4| Viral coat protein (CP) accumulation in *NbEILP*-knockdown plants. Western blot analysis of CP accumulation in knockdown plants (gray bar) inoculated with (A) *Potato virus X* (PVX) or (B) *Cucumber mosaic virus* (CMV). Levels in control plants (Luc; black bar) at 5 days post inoculation (dpi) were set to 100%. The numbers shown above each bar are the mean relative levels of CP with the standard error derived from at least 3 independent experiments. rbcL, Rubisco large subunit (loading control for normalization).
